# Uremic toxins removal and iron status: a medium-term comparison between 4 dialysis techniques (EMPIRE study)

**DOI:** 10.1080/0886022X.2025.2497491

**Published:** 2025-05-05

**Authors:** Marianna Napoli, Gaetano Alfano, Anna Scrivo, Fulvia Zappulo, Niccolò Morisi, Laura Martano, Silvia Giovanella, Marco Ferrarini, Gaetano La Manna, Gabriele Donati

**Affiliations:** ^a^ Nephrology, Dialysis and Kidney Transplant Unit, IRCCS Azienda Ospedaliero-Universitaria di Bologna, Bologna, Italy; ^b^ Department of Medical and Surgical Sciences (DIMEC), Alma Mater Studiorum-University of Bologna, Bologna, Italy; ^c^ Nephrology Dialysis and Kidney Transplant Unit, Azienda Ospedaliero-Universitaria di Modena, Modena, Italy; ^d^ Surgical, Medical, Dental and Morphological Sciences Department (CHIMOMO), University of Modena and Reggio Emilia, Modena, Italy

**Keywords:** Uremic toxins, TSAT, HFR–aequilibrium, HDx, HF-HD, OL-HDF

## Abstract

**Background:**

Recent evidence documented that dialyzers and hemodialytic techniques yield different dialytic performances. The study aims to compare uremic toxins removal and iron status between on-line hemodiafiltration (OL-HDF), high-flux hemodialysis (HF-HD), expanded hemodialysis (HDx), and HFR Aequilibrium (HFR-Aeq).

**Methods:**

A single-center retrospective observational study enrolled 52 patients on chronic HD. Each study group (HFR-Aeq, HDx, HF-HD, and OL-HDF) included 13 patients. Naïve patients for each of the treatments were considered. Serum samples were collected at baseline and after 12–24–48 weeks from the enrollment. Intragroup comparison was performed using Friedman’s test whereas longitudinal data were compared using linear mixed models (LMMs).

**Results:**

HDx showed a progressive improvement in the removal of urea (*p* = 0.043), λ -free light chains (*p* = 0.033), and transferrin saturation (*p* = 0.011) compared to other techniques. A nearly significant slope of β2 M was observed (*p* = 0.066). Also HFR-Aeq showed a near significant reduction in λ FLC values (*p* = 0.05) and a nearly significant increase in albumin levels (*p* = 0.07).

**Conclusions:**

HFR-Aeq provides uremic toxins removal comparable to other traditional techniques (HF-HD, OL-HDF). HDx confirmed its superiority in the removal of uremic toxins as urea and λ FLC and surprisingly enhanced TSAT by a possible anti-inflammatory effect not ascertained in the present study. The utilization of non-optimal convective volumes likely vanishes the promising findings of OL-HDF.

## Background

Over the years, the retention of uremic toxins has been largely investigated by studies aiming to enhance the performance of dialysis techniques and dialyzer membrane technology. Significant improvements have been achieved with on-line hemodiafiltration (OL-HDF) in terms of increased survival and improved quality of life thanks to the use of automatic ultrafiltration control systems and ultrapure dialysis fluid production [1,2]. However, application of OL-HDF is not enough to improve the survival of patients with kidney failure. Delivery of high convection volume (>23 liters) is a critical condition in determining survival advantage compared with HF-HD. Achieving such convective flows requires an efficient vascular access that allows blood flows greater than 300 mL/min or a duration of dialysis treatment greater than 4 h [3]. These conditions limit the widespread application of the OL-HDF technique. In the case of patients who are not suitable for OL-HDF due to the difficulty in obtaining efficient vascular access or due to clinical contraindications, the patients can receive HFR, HDx, and HF-HD [4]. In particular, when OL-HDF is not feasible for non-suitable vascular access, HDx or HF-HD are mandatory, in the case of patients with intradialytic hypotension where OL-HDF is ineffective, the use of HFR Aequilibrium (HFR-Aeq) allows a sodium and ultrafiltration profiling controlled by a mathematical model [5,6].

Aim of the study was the medium-term comparison of HFR, HDx, HF-HD, and OL-HDF in terms of removal of uremic toxins, improvement of inflammatory markers, and iron status in HD patients.

## Methods

A single-center, retrospective, spontaneous observational study was carried out in 52 chronic HD patients. Four groups (HFR, HDx, HF-HD, and OL-HDF) were formed and each group included 13 patients. All the patients underwent dialysis at the dialysis Center of the Nephrology Dialysis and Renal Transplantation Unit, S. Orsola University Hospital, Bologna Italy.

### Patient selection

Each patient was treated consecutively with the same dialysis technique for 48 weeks. Each of the patients enrolled had not previously undergone the treatment analyzed.

The common inclusion criteria for all the treatments were: age ≥ 18 years; chronic hemodialysis treatment (on dialysis for at least 6 months), naïve on hemodialysis treatment with HDx, HF-HD, OL-HDF, and HFR-Aeq; continuous use of the same hemodialysis treatment for 48 weeks; diuresis < 200 mL/day; thrice weekly hemodialysis treatment; session length ≥ 210 min; arteriovenous fistula, graft, or central venous catheter with blood flow rate (QB) ≥ 250 mL/min, acquisition of the informed consent.

In the case of HFR-Aeq two additional criteria were considered: intradialytic hypotension or disequilibrium syndrome as described elsewhere [6]. Patients assigned to HFR-Aeq were hypotension prone patients (>30% of dialysis session complicated by intradialytic hypotension) [7]. In summary, intradialytic hypotension was defined according to the criteria of Colì et al. [8]: (a) patients with SBP > 100 mmHg at dialysis initiation that during dialysis is reduced <90 mmHg; (b) patients with a reduction in SBP ≥ 25% associated with hypotensive symptoms regardless of dialysis initiation pressure; (c) patients with SBP < 90 mmHg at dialysis initiation with a reduction in blood pressure during dialysis ≥ 10% associated with hypotensive symptoms.

The exclusion criteria were: need for heparin-free dialysis, active bleeding; active hematological diseases; thrombocytopenia; chronic liver disease; active systemic inflammatory diseases; uncontrolled diabetes mellitus; temporary vascular access; recurrent vascular access infections; lack of clinical data.

Patients enrolled in this study were part of a larger cohort of 251 patients on chronic maintenance dialysis, the period of the study was between 2022 and 2024. Patients with a length of hospital stay <30 days were selected to avoid potential confounding factors due to acute illness. To ensure homogeneity among groups, we maintained the same number of patients between groups taking into account that the smallest group was composed of 13 subjects. When the number of patients was >13 we selected the first 13 of the list.

### Dialysis techniques and dialyzer membrane technology

For HFR-Aeq technique, a double chamber filter called HFR 17^®^ was used (Bellco, Mirandola, Italy). The first part of the filter consisted of a polyphenylene high flux filter with a coefficient of ultrafiltration (KUF) of 28 mL/h/mmHg, a surface area of 0.7 m^2^, and a membrane cutoff value of 25 kDa. The first filter produces automatically an endogenous ultrafiltrate. The ultrafiltrate is driven from this hemofilter to 40 g neutral styrene resin. After the adsorption, the ultrafiltrate is added to the whole blood that, in its turn, passes into a second polyphenylene low-flux filter (KUF 13 mL/h/mmHg, surface area 1.7 m^2^) where the weight loss and the diffusive depuration take place. The Aequilibrium configuration includes a sodium sensor called Natrium combined with a mathematical model for processing sodium and ultrafiltration profiles and maintain a neutral sodium balance during dialysis. Prescribing with HFR-Aeq is also recommended for patients with a lower albumin value due to the absence of albumin loss with this dialysis technique.

HDx was performed using single-pass bicarbonate dialysis setting with a medium cutoff (MCO) filter called Theranova^®^ 400 (Baxter, Heichingen, Germany): polyaryletheresulfone-polyvinilpirrolidone membrane, the surface area is 1.7 m^2^, steam sterilization, the KUF is 48 mL/h/mmHg, membrane cutoff is 25 kDa, pore radius 4.7–5.0 nm.

OL-HDF technique was carried out by means of FX80 Cordiax^®^ dialyzer with automatic biofeedback driven convective volume prescription (Helixone^®^, KUF 64 mL/h/mmHg, surface area 1.8 m^2^, membrane cutoff 30 kDa, Fresenius Medical Care, Bad Homburg, Germany). The reinfusion volume was administered in post-dilution with a filtration fraction between 25% and 30%.

HF-HD was performed by Revaclear^®^ 400 dialyzer was used (polyaryletheresulfone-polyvinilpirrolidone membrane, Baxter, Heichingen, Germany) with a surface area of 1.7 m^2^, membrane cutoff value 14 kDa, and KUF of 54 mL/h/mmHg).

### Data collection

Laboratory tests were collected at baseline (T0) and after 12 weeks (T12) 24 weeks (T24) and 48 weeks (T48).

Data collected included: gender, age; dialysis vintage, clinical history, the type of vascular access for hemodialysis, dialysis session length, dry weight, blood flow (QB), weekly EPO dose, weekly i.v. iron dose and Charlson Comorbidity Index (CCI). The erythropoietin resistance index (ERI) was also calculated and defined as the weekly weight-adjusted EPO dose (U/kg/week) divided by the hemoglobin level. The blood drawn was carried out before starting the first dialysis session of the week but after at least 1 weeks from the last iron administration. KT/V was measured by means of the instrumentation embedded in the dialysis machines and reported in the patients’ charts. EPO administered was epoetin alfa (Binocrit^®^, Sandoz, Milan, Italy). Iron administered was ferric gluconate (Ferlixit^®^; Sanofi, Milan, Italy) or ferric carboxymaltose (Ferinject^®^, Vifor Pharma, Rome, Italy).

The patients’ medical records were retrospectely reviewed and the percentage of dialysis sessions with symptomatic hypotension were recorded. This percentage was calculated not on the single day when the blood tests were taken, but during the month before considering the 122 dialysis sessions that the 13 patients performed in the HFR-Aeq group. The percentage of dialysis sessions free of intradialytic hypotension in the month before starting T0 (1mbT0), the month before T12 (1mbT12), the month before T24 (1mbT24), and the month before T48 (1mbT48) was compared.

Low molecular weight heparin enoxaparin (Clexane^®^, Sanofi, Milan Italy) was used for the anticoagulation of the extracorporeal circuit. A dose of 2000 IU (patients < 50 kg of body weight), 4000 IU (patients between 50 to 90 kg of body weight), or 6000 IU (patients > 90 kg of body weight) was administered in a single bolus on starting dialysis [9]. The QB rate from the vascular access was ≥250 mL/min. The dialysate flow rate was 500 mL/min.

The concentrations of κ and λ free light chains (FLC) and of beta 2 microglobulin (β2M) were measured by nephelometry (kit Freelite k/lambda, The Binding Site Greey, Birmingham, UK; IIMAGE/IMMAGE 800 Beckman Coulter instrument, Brea California USA, Beckman Coulter β2M kit). Normal values: κ FLC 3.3 − 19.9 mg/L, λ FLC 5.7–26.3 mg/L, β_2_M 0.7 − 2.0 mg/L. The concentration of ferritin was measured by means of chemiluminescence-immunoassay, normal values: 11–336 ng/mL. The concentration of C-reactive protein (CRP) was measured by turbidimetry (CRPLX, Tina-quant C-Reactive Protein; Roche/Hitachi 902 analyzer). CRP normal value <0.8 mg/dL. Molecular weights: β2M 12 kDa, κ FLC 22.5 kDa; λ FLC 45 kDa, CRP 120 kDa, ferritin 474 kDa. Albumin, hemoglobin, urea, creatinine, phosphates, and the other laboratory tests were assessed using the common laboratory methods.

#### Statistical analysis

Normality of continuous data was explored histographically and verified using the Shapiro- Wilk test and the Kolmogorov-Smirnov. In this study, non-normally distributed data were presented as median and interquartile range (IQR; 25%–75%). Categorical variables were reported as count and percentage.

Kruskal-Wallis test has been used for comparisons of baseline data between the four groups whereas comparisons for categorical data were made using χ^2^ or Fisher exact test when contingency table cell counts were < 5. We explored within-subject differences over different time points (0, 12, 24, 48 weeks) through the application of Friedman’s test (a non-parametric alternative of the ANOVA repeated measures).

Linear mixed model was used to analyze longitudinal data of HF-HD, HFR-Aeq, OL-HDF, and HDx groups at four visits over 48 weeks. The models included the two-way interaction between dialysis modality and time, random intercept, and random slope with an unstructured variance-covariance matrix. Linear mixed models were run unadjusted and adjusted, with the latter model including the following covariates: age, sex, weight, albumin, time of dialysis treatment, and Qb of vascular access. To avoid over-adjustment bias, we carefully selected each confounder based on their established causal relationship with the outcome. The results of the linear mixed analysis reported the overall weekly variation of the outcome variables for HFR-Aeq, OL-HDF, and HDx groups. These values were compared with HF-HD, the reference group in our study.

The SPSS v26 (Armonk, NY, USA) software program was used for descriptive statistical analysis whereas STATA v.14 (College Station, TX, USA) was used to perform linear mixed model analysis.

## Results

Fifty-two patients were considered for the present study. Among them 65.4% are men and the median age was 70.5 years (55–77.8). Overall, patients were affected by diabetes mellitus (44.3%), arterial hypertension (57.7%), and cardiovascular disease (75%). The demographic and clinical characteristics of the patients enrolled in the four groups are shown in Table 1. The four groups are homogeneous in terms of age, sex, and ethnicity. The patients did not differ in comorbidities except for arterial hypertension. As expected, the patients who underwent HFR-Aeq were affected by intradialytic hypotension. Laboratory parameters at baseline are reported in Table 2. The four groups differed only in baseline albumin values. In particular, patients who had been prescribed HFR-Aeq had the lowest albumin values among the four groups (*p* = 0.001).

**Table 1.** Patients’ characteristics.

	HFR-Aeq	HDx	OL-HDF	HF-HD	*p*
Patients (*n*)	13	13	13	13	*-*
Age (years)	66 (56–74)	67 (49.5–76)	77(68–83)	63 (44.5–77)	0.12
Males (%)	38.5	30.8	38.5	30.8	0.95
Caucasian %	84.6	100	84.6	92.3	0.81
Dry weight (kg)	62 (51.7–89.8)	74.8 (70.5–77.3)	67 (56.8–82.8)	60.3 (50–69.3)	0.09
Vascular access %					**0.03**
AVF	69.2	100	84.6	53.8	
CVC	30.8	0	15.4	46.2	
Comorbidities %					
DM	30.8	53.8	53.8	38.5	0.55
Hypertension	0	84.6	69.2	69.2	**<0.0001**
CVD	61.5	84.6	92.3	61.5	0.16
Previous cancers	15.4	23.1	30.8	23.1	0.83
Obesity	23.1	7.7	15.4	15.4	0.76
COPD	23.1	7.7	15.4	7.7	0.61
CCI score	5 (4–7)	5 (3.5–8)	8 (6–9)	6 (3–9)	0.14
Dialysis duration (months)	65 (50.5–107)	77 (54–105)	104 (20–149.5)	53 (45.5–167.5)	0.934
Hospitalization days/48 week	5 (0.5–20)	5 (3–12.5)	15 (3–28)	9 (4.5–25)	0.406
KT/V	1.53 (0.96–1.76)	1.39 (1.19–1.55)	1.39 (1.35–1.65)	1.37 (1.28–1.65)	0.91
URR	0.72 (0.54–0.8)	0.7 (0.65–0.74)	0.7 (0.6–0.75)	0.7 (0.66–0.74)	0.90
QB, mL/min	300 (280–320)	300 (280–300)	300 (265–320)	280 (250–300)	0.41

HDx, expanded hemodialysis; HFR, hemodiafiltration with reinfusion of endogenous ultrafiltrate; OL-HDF, online hemodiafiltration; HF-HD, high flux hemodialysis; AVF, arteriovenous fistula; CVC, central venous catheter; DM, Diabetes mellitus; CVD, cardiovascular diseases; COPD, Chronic obstructive pulmonary disease; CCI, Charlson comorbidity index; K, urea clearance; T, time of dialysis; V, urea distribution volume; URR, urea reduction rate; QB, blood flow.

Bold indicates best results.

**Table 2.** Differences in laboratory parameters at baseline between groups.

	HFR-Aeq	HDx	OL-HDF	HF-HD	*p*
Urea (mg/dL)	129 (98–149.5)	145 (134–186)	144 (103.5–159)	158 (129.5–177.5)	0.09
Creatinine (mg/dL)	8.7 (7.1–10.2)	9.4 (8.2–11.6)	8.1 (5.1–10.6)	8.1 (6.5–9.9)	0.33
Phosphates (mg/dL)	4.8 (4.5–5.6)	6.0 (4.6–6.8)	4.6 (3.7–6.0)	4.8 (3.8–6.0)	0.29
β2-microglobulin (mg/L)	32.6 (30–36.9)	30.8 (27.4–39.8)	31 (26.1–34.9)	30.4 (18.8–35.4)	0.46
κ FLC (mg/L)	168.8 (140.2–218.8)	176.3 (135.3–225)	118.4 (97.8–260.3)	112.6 (80.1–169.25)	0.08
λ FLC (mg/L)	98.7 (79.5–173.6)	110.0 (72.7–142.2)	121.9 (91.3–127.6)	91 (61.3–134.6)	0.69
Albumin (gr/dL)	3.4 (3.1–3.6)	3.8 (3.6–3.9)	3.5 (3.1–3.7)	3.8 (3.5–4)	**0.001**
Hb (gr/dL)	10.1 (9.7–11.6)	10.5 (9.8–11.9)	10.4 (9.4–11.4)	11.1 (10.1–11.9)	0.64
Ferritin (ng/dL)	123 (74.5–231.5)	128 (44.5–229)	228 (85.5–417)	113 (43–349.5)	0.59
Transferrin (mg/dL)	209 (145.5–244.5)	175 (172–207)	183 (147.5–211)	205 (157.5–241)	0.60
TSAT (%)	16.5 (13.3–29.9)	16.5 (13–25)	20 (7.6–26.5)	23 (13.7–39.1)	0.75
Iron dose (mg/week)	100 (31.3–200)	62.5 (31.3–62.5)	0 (0–143)	0 (0–187.5)	0.71
ERI	17.9 (10–34.9)	24.3 (14.4–32.8)	21.6 (13.5–46.3)	12.5 (0–62.6)	0.65
CRP (mg/dL)	0.8 (0.3–1.3)	0.3 (0.1–1.3)	0.6 (0.2–1.8)	0.34 (0.11–1.1)	0.35

The values are referred to the blood drawn on starting the first dialysis considered for the study. The data are reported as median and interquartile range.

FLC, free light chains; Hb, hemoglobin; TSAT, transferrin saturation; ERI; Erythropoietin resistance index; CRP, C-reactive protein.

Bold indicates best results.

During the follow-up, patients had a median of 2 (1–4) episodes of hospital admission and length of stay was 8 (3–19) days. No statistically significant differences were found in terms of hospital admission (*p* = 0.825) and length of stay (*p* = 0.406) between groups.

To ascertain the risk of a carry-over effect due to the previous type of dialysis treatment, the results obtained at T0 were compared with the results obtained at T12 in each group. No differences were found between the two sampling times to detect a carry-over effect (Table 1 Supplementary Material).

### Intra-group differences

#### HFR-Aeq

The efficacy of HFR-Aeq in removing uremic toxins is detailed in Table 2 Supplementary Material. Data analysis shows that there was no significant variation in the removal of these molecules, nor in the martial metabolism and ERI. It is important to point out that there was an increase in blood albumin values during the study period. In particular, albumin increases from 3.4 gr/dL (3.1–3.5) at T0 to 3.6 gr/dL (3.3–3.8) at T24.

**Figure 1.**
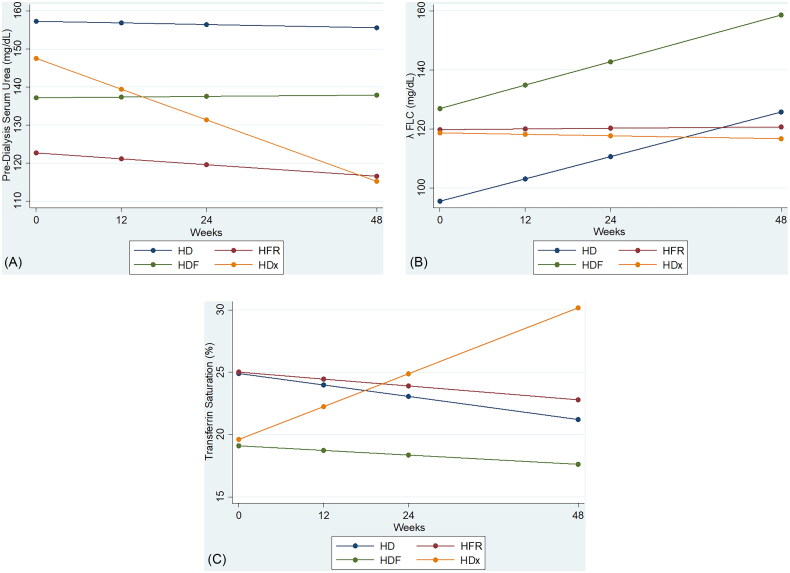
Slope estimates of urea (a), λ FLC (b), transferrin saturation (c), based on the analysis of longitudinal data by linear mixed effects models.

As to the percentage of HFR-Aeq sessions free of intradialytic hypotension in the month before T0, it was significantly lower than the percentage of HFR-Aeq sessions free of intradialytic hypotension during the follow-up period (*p* = 0.039, Figure 1 Supplementary Material).

#### HDx

Treatment with HDx led to a significant decrease in urea level during all 4-blood test timepoints (Table 3, Supplementary Material). Median values of urea at baseline decreased from 145 mg/dL (134.5–186) to 122 mg/dL (105.5–139) after 48 weeks, *p* = 0.016. No difference was observed in Qb, dialysis time, and KT/V. As far as the iron balance is concerned, a significant increase in the TSAT was observed despite a stable dose of iron: TSAT increased from 16.5% (13–25) to 25.5% (23–30.2). Transferrin value remained nearly stable from T0 (175 mg/dL [172–207]) to T48 (170 mg/dL [140–190]) whilst ERI decreased with a non-significant trend from 25.2 (14.9–32.7) to 6.5 (0.6–37.7) (*p* = 0.56).

#### OL-HDF

Patients treated with OL-HDF experienced a progressive and significant increase in β2 M without significant retention of the other uremic toxins and without significant changes in KT/V and blood flow (Table 4, Supplementary Material). β2 M increased from 31 mg/L (26.1–34.9) at T0 to 38.5 mg/L (33.4–43.6) at T48 (*p* = 0.002). No significant variations in iron metabolism and ERI were observed over the four timepoints.

#### HF-HD

Treatment with HF-HD did not lead to a significant variation in the removal of uremic toxins and did not improve iron metabolism and ERI (Table 5, Supplementary Material).

#### Between groups differences at the end of the follow-up

Considering that the four groups exhibited homogeneity, except for albumin, in baseline variables, a comparison was conducted to identify differences in uremic toxin removal and ERI variation after 48 weeks of treatment with the different dialysis modalities.

#### HDx vs. OL-HDF

Dialysis treatment using a medium cutoff membrane significantly improved dialysis efficiency compared to OL-HDF.

Urea level was significantly lower in the HDx group (122 mg/dL [105–139]) compared to OL-HDF (158 mg/dL [149 – 191]) (*p* = 0.02).

The median pre-dialysis β2 M in HDx measured 31.2 mg/dL (27.8–36.7) compared 38.5 mg/dL (33.5–43.6) in OL-HDF. This difference was statistically significant (*p* = 0.029).

An increase at T48 in TSAT levels was observed in HDx group compared with OL-HDF group: 25.5% (23–30.2) vs 11.4% (9.2–22.7) (*p* = 0.007).

#### HFR-Aeq vs. OL-HDF

A significant reduction of iron supplementation was observed in HFR-Aeq (187.5 mg/week [50–300]) vs. OL-HDF group (62.5 mg/week [0–112.5]) at T48 (*p* = 0.049). HFR-Aeq also determined a non-statistical significant reduction of the smallest FLC. Indeed, median κ FLC measured 146.1 mg/L (115.0–189.7) in HFR-Aeq group compared 189.3 mg/L (140.1–280.1) in OL-HDF (*p* = 0.077).

#### HFR-Aeq vs. HDx

TSAT levels reached a significantly high value in HDx (25.5% [23–30.2]) when compared to HFR-Aeq (19.7[14.2–26.6]) (*p* = 0.04). There was also a reduction of the ERI, iron, and EPO dose in HDx group during the observation period but without a statistical significance when compared with HFR-Aeq.

No other significant differences between the four groups were observed throughout the study in terms of low- and middle-molecular-weight toxins removal.

#### Linear mixed models

The longitudinal data analysis of HF-HD, HFR-Aeq, OL-HDF, and HDx groups at four visits over 48 weeks was carried out by means of linear mixed models (Tables 3 and 4). The analysis showed a significant reduction of urea values with HDx use (*p* = 0.043) when adjusted for age, sex, weight, albumin, time of dialysis treatment, and Qb of vascular access. Again, in the adjusted analysis, HDx has been shown to significantly reduce λ FLC values (*p* = 0.033) and to significantly increase TSAT values (*p* = 0.011), nonetheless a nearly significant slope of β2 M was observed (*p* = 0.066) (Tables 3 and 4 and Figure 1). Also HFR-Aeq, in the adjusted analysis, showed a near significant reduction in λ FLC values (*p* = 0.05) and a nearly significant increase in albumin levels (*p* = 0.07) (Tables 2 and 3). As to kappa FLC, ERI, phosphorus, CRP, and Hb values no differences were found (Table 4).

**Table 3.** The slope over time of the outcomes calculated by linear mixed effects model (urea, λ FLC, TSAT).

	Unadjusted				Adjusted			
	Urea slope (mg/dL/week)	Urea difference with HF-HD (mg/dL/week)	*p* value	95% CI	Urea slope (mg/dL/week)	Urea difference with HF-HD (mg/dL/week)	*p* value	95% CI
HF-HD	(ref.)	(ref.)	(ref.)	(ref.)	(ref.)	(ref.)	(ref.)	(ref.)
HFR-Aeq	−0.07	0.03	0.9	−0.51 − 0.58	−0.07	−0.01	0.96	0.58 − 0.55
OL-HDF	0.06	0.17	0.52	−0.37 − 0.72	0.04	0.1	0.7	0.45 − 0.67
HDx	−0.66	−0.56	**0.043**	−1.11 − −0.017	−0.63	−0.57	**0.043**	−1.13 − −0.01
	Unadjusted				Adjusted			
	λ FLC slope (mg/dL/week)	λ FLC difference with HF-HD (mg/dL/week)	*p* value	95% CI	λ FLC slope (mg/dL/week)	λ FLC difference with HF-HD (mg/dL/week)	*p* value	95% CI
HF-HD	(ref.)	(ref.)	(ref.)	(ref.)	(ref.)	(ref.)	(ref.)	(ref.)
HFR-Aeq	−0.122	−0.702	**0.02**	−1.3 − −0.1	0.01	−0.61	**0.05**	−1.2 − 0.01
OL-HDF	0.579	−0.001	0.96	−0.59 − 0.59	0.65	0.03	0.92	−0.58 − 0.64
HDx	−0.07	−0.65	**0.034**	−1.25 − −0.05	0.05	−0.67	**0.033**	−1.28 − −0.05
	Unadjusted				Adjusted			
	*TSAT* slope (% - week)	TSAT difference with HF-HD (% - week)	*p* value	95% CI	*TSAT* slope (% - week)	TSAT difference with HF-HD (% - week)	*p* value	95% CI
HF-HD	(ref.)	(ref.)	(ref.)	(ref.)	(ref.)	(ref.)	(ref.)	(ref.)
HFR-Aeq	−0.05	0.04	0.72	−0.18 − 0.26	−0.04	0.03	0.78	−0.18 − 0.25
OL-HDF	−0.07	0.02	0.80	−0.19 − 0.25	−0.03	0.04	0.68	−0.17 − 0.26
HDx	0.22	0.31	**0.008**	0.08 − 0.54	0.22	0.29	**0.011**	0.06 − 0.52

HFR-Aeq, HFR Aequilibrium; OL-HDF, online hemodiafiltration; HDX, expanded hemodialysis; HF-HD, high flux hemodialysis; FLC, free light chains; TSAT, transferrin saturation.

Bold indicates best results.

**Table 4.** The slope over time of the outcomes calculated by linear mixed effects model (β2 M, kappa FLC, ERI, albumin, phosphorus, CRP, and Hb).

	Unadjusted				Adjusted			
	β2-M slope (mg/dL/week)	β2-M difference with HF-HD (mg/dL/week)	*p* value	95% CI	β2-M slope (mg/dL/week)	β2-M difference with HF-HD (mg/dL/week)	*p* value	95% CI
HF-HD	(ref.)	(ref.)	(ref.)	(ref.)	(ref.)	(ref.)	(ref.)	(ref.)
HFR-Aeq	−0.01	−0.08	0.15	−0.2 − 0.03	−0.01	−0.09	0.1	−0.2 − 0.01
OL-HDF	0.15	0.07	0.210	−0.04 − 0.19	0.1	0.02	0.61	−0.82 − 0.13
HDx	−0.02	−0.097	0.117	−0.21 − 0.02	−0.02	−0.1	0.066	−0.21 − 0.01
	Unadjusted				Adjusted			
	κ FLC slope (mg/dL/week)	κ FLC difference with HF-HD (mg/dL/week)	*p* value	95% CI	κ FLC slope (mg/dL/week)	κ FLC difference with HF- HD (mg/dL/week)	*p* value	95% CI
HF-HD	(ref.)	(ref.)	(ref.)	(ref.)	(ref.)	(ref.)	(ref.)	(ref.)
HFR-Aeq	−0.46	−1.02	0.107	−2.26 − 0.21	−0.4	−1.06	0.09	−2.3 − 0.2
OL-HDF	0.87	0.31	0.61	−0.91 − 1.54	0.82	0.16	0.79	−1.07 − 1.4
HDx	−0.29	−0.85	0.18	−2.1 − 0.39	−0.18	−0.84	0.18	−2.08 − 0.4
	Unadjusted				Adjusted			
	ERI slope (% - week)	ERI difference with HF-HD (% - week)	*P* value	95% CI	ERI slope (% - week)	ERI difference with HF-HD (% - week)	*p* value	95% CI
HF-HD	(ref.)	(ref.)	(ref.)	(ref.)	(ref.)	(ref.)	(ref.)	(ref.)
HFR-Aeq	0.05	−0.02	0.92	−0.45 − 0.40	0.066	−0.031	0.88	−0.45 − 0.38
OL-HDF	**0.04**	−0.03	0.88	−0.46 − 0.39	0.082	−0.015	0.94	−0.43 − 0.40
HDx	−0.09	−0.16	0.44	−0.6 − 0.26	−0.083	−0.18	0.39	−0.60 − 0.24
	Unadjusted				Adjusted			
	Albumin slope (gr/dL/week)	Albumin difference with HF-HD (gr/dL/week)	*p* value	95% CI	Albumin slope (gr/dL/week)	Albumin difference with HF-HD (gr/dL/week)	*p* value	95% CI
HF-HD	(ref.)	(ref.)	(ref.)	(ref.)	(ref.)	(ref.)	(ref.)	(ref.)
HFR-Aeq	0.002	0.003	0.15	−0.001 − 0.009	0.0026	0.004	0.07	−0.0005 − 0.0102
OL-HDF	−0.002	−0.001	0.64	−0.006 − 0.004	−0.0024	−0.001	0.63	−0.006 − 0.004
HDx	−0.001	−0.0001	0.96	−0.005 − 0.005	−0.0013	0.0001	0.95	−0.005 − 0.005
	Unadjusted				Adjusted			
	Phosphorus slope (gr/dL/week)	Phosphorus difference with HF-HD (gr/dL/week)	*p* value	95% CI	Phosphorus slope (gr/dL/week)	Phosphorus difference with HF HD (gr/dL/week)	*p* value	95% CI
HF-HD	(ref.)	(ref.)	(ref.)	(ref.)	(ref.)	(ref.)	(ref.)	(ref.)
HFR-Aeq	0.009	−0.003	0.8	−0.03 − 0.02	0.009	−0.006	0.66	−0.03 − 0.02
OL-HDF	0.011	−0.001	0.92	−0.03 − 0.02	0.007	−0.008	0.57	−0.03 − 0.02
HDx	−0.008	−0.02	0.1	0.05 − 0.004	−0.008	−0.023	0.11	−0.05 − 0.005
	Unadjusted				Adjusted			
	CRP slope (gr/dL/week)	CRP difference with HF-HD (gr/dL/week)	*p* value	95% CI	CRP slope (gr/dL/week)	CRP difference with HF HD (gr/dL/week)	*p* value	95% CI
HF-HD	(ref.)	(ref.)	(ref.)	(ref.)	(ref.)	(ref.)	(ref.)	(ref.)
HFR-Aeq	−0.0025	−0.003	0.98	−0.03 − 0.03	−0.155	−0.004	0.81	−0.03 − 0.04
OL-HDF	−0.0068	−0.004	0.78	−0.03 − 0.02	−0.168	−0.008	0.67	−0.04 − 0.02
HDx	0.0047	0.007	0.68	−0.02 − 0.04	0.153	0.007	0.68	−0.02 − 0.04
	Unadjusted				Adjusted			
	Hb slope (gr/dL/week)	Hb difference with HF-HD (gr/dL/week)	*p* value	95% CI	Hb slope (gr/dL/week)	Hb difference with HF HD (gr/dL/week)	*p* value	95% CI
HF-HD	(ref.)	(ref.)	(ref.)	(ref.)	(ref.)	(ref.)	(ref.)	(ref.)
HFR-Aeq	−0.002	−0.014	0.37	−0.04 − 0.01	−0.003	−0.017	0.26	−0.03 − 0.01
OL-HDF	0.004	−0.008	0.61	−0.04 − 0.02	0.008	−0.006	0.66	−0.03 − 0.02
HDx	0.013	0.001	0.94	−0.03 − 0.03	0.015	0.0008	0.95	−0.02 − 0.03

HFR-Aeq, HFR Aequilibrium; OL-HDF, online hemodiafiltration; HDX, expanded hemodialysis; HF-HD, high flux hemodialysis; FLC, free light chains; β2 M, beta 2 microglobulin; ERI, Erythropoietin resistance index; CRP, C-reactive protein; Hb, hemoglobin.

Bold indicates best results.

## Discussion

A retrospective study comparing HFR-Aeq to OL-HDF, HF HD, and HDx during a 48-week follow-up was carried out to assess any difference in terms of uremic toxin removal, improvement of inflammatory markers, and ERI between these four techniques. At the best of our knowledge, this is the first study where these techniques were compared during a 48-week follow-up with HFR-Aeq. The presence of intradialytic hypotension and hypoalbuminemia justify the prescription of HFR-Aeq and the incomplete propensity score matching between HFR-Aeq and other dialysis techniques.

The main findings of the study are summarized in the following points: (a) HFR-Aeq performed in hypotension-prone patients did not show any differences in uremic toxins removal and iron status in comparison with OL-HDF and HF-HD performed in non-hypotension-prone HD patients; (b) OL-HDF did not show any improvement in uremic toxins removal and ERI in comparison to the other dialysis techniques when nonoptimal convective volumes were achieved; (c) HDx showed a progressive improvement in urea removal, lambda FLC removal and TSAT, but not in ERI, in comparison to the other dialysis techniques.

### HFR-Aeq

Hypotension-prone patients experience a high rate of mortality and morbidity, including an augmented rate of vascular access thrombosis and a reduced dialysis dose, in comparison to non-hypotension-prone dialysis patients [10–13]. In this context, combined with intradialytic hypotension, HFR-Aeq was prescribed due to its well-known capacity for negligible intra-dialytic amino acid loss and absence of albumin loss [14]. When between-group differences were considered, our study did reveal significant differences of λ FLC between the four groups with a nearly better slope of λ FLC with HFR-Aeq in mixed models analysis. According to the initial studies assessing HFR performance, this technique demonstrated superiority over low-flux hemodialysis and non-inferiority compared to OL-HDF [15]. In the last years, comparisons were carried out between OL-HDF and the last HFR configuration called SUPRA HFR with an increased amount of resin suitable for toxins adsorption [16]. In this case, despite the convective volume of OL-HDF was not adjusted to the patients’ body surface area, SUPRA improved uremic protein-bound toxins levels and microinflammation [16]. Our group carried out a single session analysis between SUPRA HFR and OL-HDF, both techniques afford a significant reduction of FGF-23 and of some cytokines, but no difference was found between them [17].

#### OL-HDF

Our results confirm that no significant differences can be found applying OL-HDF without a clinically oriented convective flux as suggested by Peters et al. [18]. Particular attention should be paid to the fact that there has been a worsening of β2M values after 48 weeks in those patients who underwent OL-HDF. The utilization of non-optimal convective volumes likely vanishes the promising findings of the CONTRAST, Turkish, and ESHOL studies, which demonstrated the superiority of OL-HDF over HD when employing high convective volumes [19–21].

#### HDx

HDx has been traditionally applied for the efficient removal of large-middle molecules beyond the depurative capacity of HF-HD and OL-HDF. Surprisingly, our study highlighted a significant reduction in urea values at the start of dialysis after 48 weeks of follow-up in the group of patients on HDx compared to patients treated in the other three groups. Yang et al. performed a meta-analysis that included 529 patients where they found no differences in urea and creatinine levels between HDx and HF-HD [22]. No other study on this topic is available at present. Nonetheless, the periodic dosage of albumin levels allowed us to exclude concomitant malnutrition in the patients of the HDx group that could explain the urea reduction, in fact, there was no significant reduction in albumin during the follow-up period. As to λ FLC our study is in line with Zickler et al. who carried out a multicenter randomized controlled trial, with 172 chronic dialysis patients, and showed that the reduction of κ FLC and λ FLC was significantly higher in the HDx group than in the HF-HD group after 24 weeks [23]. It is a very important result because it is widely accepted that serum FLC levels are independently associated with morbidity in patients with ESRD [24]. Unfortunately, we were not able to demonstrate the same for κ FLC which removal should be easier given the lower molecular weight compared to the λ FLC and given that these proteins in the case of ESKD accumulate due to lack of elimination and not due to increased production [25].

In our study, we evaluated the effect of dialysis modality on the variation of TSAT, the iron status maker that predicts response to intravenous erythropoietin. Only patients treated with HDx showed a significantly improved TSAT level compared to the other groups. The Korean group of Koo et al. showed that low TSAT was a significant independent risk factor for adverse clinical outcome in 879 incident dialysis patients with anemia, which may be partly attributed to cardiac dysfunction and inflammation [26]. Nonetheless, despite the improvement in TSAT, there was no improvement in ERI. There are scarce data that compared HDx with OL-HDF as to the control of anemia, whereas in two multicenter observational studies and in one randomized controlled study, patients in the HDx groups showed significant reductions of iron dose, EPO dose, and ERI with significant increases in serum iron levels and TSAT compared to patients with HF-HD [27–29].

## Conclusion

The current study reflects the application of the four dialysis techniques observed in the real world. Its aim was to identify the main laboratory differences in actual clinical decision-making rather than establish definitive causal relationships. HFR-Aeq provided similar uremic toxins removal and a similar ERI in comparison to the other traditional techniques (HF-HD, OL-HDF). HDx was superior to all the other three dialysis techniques as to urea, λ FLC, and TSAT, without any albumin leakage. OL-HDF can be considered detrimental if a non-optimal convective volume is achieved during dialysis. The weaknesses of the study are related to the retrospective data collection, the small sample, and the limited number of uremic toxins studied. Certainly there is a selection bias based on the clinical experience of the medical staff, this related to the assignment of patients to the four dialysis technique groups. In addition, the consideration of blood draws during the first session of the week may be influenced by the accumulation of water and toxins in the long dialysis interval. However, such current sampling schedule of the dialysis center could not be changed in the present retrospective study. Nonetheless, our medium-term evaluation reflects faithfully what happens to the patient to whom a particular dialysis technique is applied.

## Supplementary Material

Table 1 Supplementary Material.docx

Table 3 Supplementary Material .docx

Table_4_Supplementary_Material.docx

Table 2 Supplementary Material.docx

Table 5 Supplementary Material.docx

## Data Availability

The datasets used and analyzed during the current study are available from the corresponding author upon reasonable request.
