# Supplementary material for: Uremic toxins removal and iron status: a medium-term comparison between 4 dialysis techniques (EMPIRE study)
Source: Ren Fail. 2025 May 5;47(1):2497491. doi: 10.1080/0886022X.2025.2497491 (PMC12054563; doi:10.1080/0886022X.2025.2497491)
Supplement: Table 1 Supplementary Material.docx [file IRNF_A_2497491_SM8555.docx]

Table 1 Supplementary Material: differences of laboratory and clinical parameters between T0 and T12.

| **Dialysis technique** | **Variable** | **Time 0  (median)** | **Time 12 (median)** | **Difference  T12-T0** | **P value** |
| --- | --- | --- | --- | --- | --- |
| **HDx** | Albumin (gr/dL) | 3.75 | 3.58 | -0.17 | 0.09 |
| **HDx** | b2-microglobulin (mg/L) | 30.75 | 29.55 | -1.20 | 0.34 |
| **HDx** | Creatinine (mg/dL) | 9.35 | 9.70 | 0.35 | 0.68 |
| **HDx** | ERI | 24.79 | 18.48 | -6.31 | 0.97 |
| **HDx** | Erythropoietin (UI/week) | 18000.00 | 14000.00 | -4000.00 | 1.00 |
| **HDx** | Ferritin (ng/dL) | 128.00 | 122.00 | -6.00 | 0.27 |
| **HDx** | Hb (gr/dl) | 10.60 | 10.25 | -0.35 | 0.62 |
| **HDx** | Iron dose (mg/week) | 62.50 | 62.50 | 0.00 | 0.10 |
| **HDx** | k-FLC (mg/L) | 176.30 | 187.00 | 10.70 | 0.09 |
| **HDx** | KT/V | 1.39 | 1.33 | -0.06 | 0.45 |
| **HDx** | λ -FLC (mg/L) | 110.00 | 115.10 | 5.10 | 0.90 |
| **HDx** | CRP (mg/dL) | 0.29 | 0.17 | -0.12 | 0.79 |
| **HDx** | Dry weight (Kg) | 74.80 | 75.00 | 0.20 | 1.00 |
| **HDx** | Phosphates (mg/dL) | 6.00 | 5.80 | -0.20 | 0.95 |
| **HDx** | Protein (gr/dl) | 6.10 | 6.10 | 0.00 | 0.41 |
| **HDx** | QB (ml/min) | 300.00 | 300.00 | 0.00 | 0.85 |
| **HDx** | Transferrin (mg/dL) | 175.00 | 176.00 | 1.00 | 0.12 |
| **HDx** | Treatment time (min) | 240.00 | 240.00 | 0.00 | 0.32 |
| **HDx** | TSAT (%) | 16.50 | 17.40 | 0.90 | 0.37 |
| **HDx** | Urea (mg/dL) | 145.00 | 135.00 | -10.00 | 0.03 |
|  |  |  |  |  |  |
| **OL-HDF** | Albumin (gr/dL) | 3.50 | 3.60 | 0.10 | 0.22 |
| **OL-HDF** | b2-microglobulin (mg/L) | 31.00 | 35.00 | 4.00 | 0.50 |
| **OL-HDF** | Ultrafiltration (L/session) | 2.50 | 2.50 | 0.00 | 0.76 |
| **OL-HDF** | Creatinine (mg/dL) | 8.13 | 8.76 | 0.63 | 1.00 |
| **OL-HDF** | ERI | 21.59 | 8.49 | -13.10 | 0.17 |
| **OL-HDF** | Erythropoietin (UI/week) | 12000.00 | 7000.00 | -5000.00 | 0.32 |
| **OL-HDF** | Ferritin (ng/dL) | 228.00 | 200.00 | -28.00 | 0.59 |
| **OL-HDF** | Hb (gr/dl) | 10.40 | 10.90 | 0.50 | 0.27 |
| **OL-HDF** | Iron dose (mg/week) | 0.00 | 62.50 | 62.50 | 0.40 |
| **OL-HDF** | k-FLC (mg/L) | 118.40 | 149.50 | 31.10 | 0.89 |
| **OL-HDF** | KT/V | 1.39 | 1.45 | 0.06 | 0.79 |
| **OL-HDF** | λ -FLC (mg/L) | 121.90 | 112.30 | -9.60 | 0.84 |
| **OL-HDF** | CRP (mg/dL) | 0.56 | 0.57 | 0.01 | 0.54 |
| **OL-HDF** | Dry weight (Kg) | 67.00 | 67.00 | 0.00 | 0.16 |
| **OL-HDF** | Phosphates (mg/dL) | 4.60 | 4.70 | 0.10 | 0.66 |
| **OL-HDF** | Protein (gr/dl) | 6.20 | 6.30 | 0.10 | 0.54 |
| **OL-HDF** | QB (ml/min) | 300.00 | 300.00 | 0.00 | 0.65 |
| **OL-HDF** | Transferrin (mg/dL) | 183.00 | 183.00 | 0.00 | 0.20 |
| **OL-HDF** | Treatment time (min) | 240.00 | 240.00 | 0.00 | 0.32 |
| **OL-HDF** | TSAT (%) | 20.00 | 20.55 | 0.55 | 0.68 |
| **OL-HDF** | Urea (mg/dL) | 144.00 | 121.00 | -23.00 | 0.24 |
|  |  |  |  |  |  |
| **HFR** | Albumin (gr/dL) | 3.40 | 3.50 | 0.10 | 0.22 |
| **HFR** | b2-microglobulin (mg/L) | 32.60 | 36.70 | 4.10 | 0.41 |
| **HFR** | Creatinine (mg/dL) | 8.73 | 8.35 | -0.38 | 0.31 |
| **HFR** | ERI | 17.92 | 20.18 | 2.26 | 0.88 |
| **HFR** | Erythropoietin (UI/week) | 12000.00 | 12000.00 | 0.00 | 0.44 |
| **HFR** | Ferritin (ng/dL) | 123.00 | 158.00 | 35.00 | 0.79 |
| **HFR** | Hb (gr/dl) | 10.10 | 10.50 | 0.40 | 0.68 |
| **HFR** | Iron dose (mg/week) | 100.00 | 200.00 | 100.00 | 0.14 |
| **HFR** | k-FLC (mg/L) | 168.80 | 151.80 | -17.00 | 0.27 |
| **HFR** | KT/V | 1.53 | 1.30 | -0.23 | 0.68 |
| **HFR** | λ -FLC (mg/L) | 98.65 | 96.85 | -1.80 | 0.97 |
| **HFR** | CRP (mg/dL) | 0.79 | 0.50 | -0.29 | 0.84 |
| **HFR** | Dry weight (Kg) | 71.70 | 70.70 | -1.00 | 0.79 |
| **HFR** | Phosphates (mg/dL) | 4.80 | 5.20 | 0.40 | 0.91 |
| **HFR** | Protein (gr/dl) | 5.80 | 5.70 | -0.10 | 0.69 |
| **HFR** | QB (ml/min) | 300.00 | 300.00 | 0.00 | 0.67 |
| **HFR** | Transferrin (mg/dL) | 209.00 | 169.00 | -40.00 | 0.11 |
| **HFR** | Treatment time (min) | 240.00 | 240.00 | 0.00 | 0.32 |
| **HFR** | TSAT (%) | 16.49 | 21.30 | 4.81 | 0.74 |
| **HFR** | Urea (mg/dL) | 129.00 | 115.00 | -14.00 | 0.50 |
|  |  |  |  |  |  |
| **HF-HD** | Albumin (gr/dL) | 3.80 | 3.80 | 0.00 | 0.17 |
| **HF-HD** | b2-microglobulin (mg/L) | 32.45 | 33.10 | 0.65 | 0.85 |
| **HF-HD** | Creatinine (mg/dL) | 7.78 | 8.84 | 1.06 | 0.18 |
| **HF-HD** | ERI | 11.30 | 17.90 | 6.60 | 0.96 |
| **HF-HD** | Erythropoietin (UI/week) | 8000.00 | 12000.00 | 4000.00 | 0.89 |
| **HF-HD** | Ferritin (ng/dL) | 113.00 | 216.00 | 103.00 | 0.89 |
| **HF-HD** | Hb (gr/dl) | 11.10 | 11.50 | 0.40 | 0.95 |
| **HF-HD** | Iron dose (mg/week) | 0.00 | 0.00 | 0.00 | 0.48 |
| **HF-HD** | k-FLC (mg/L) | 112.60 | 116.60 | 4.00 | 0.31 |
| **HF-HD** | KT/V | 1.37 | 1.46 | 0.09 | 0.34 |
| **HF-HD** | λ -FLC (mg/L) | 91.00 | 79.70 | -11.30 | 0.84 |
| **HF-HD** | CRP (mg/dL) | 0.34 | 0.46 | 0.12 | 1.00 |
| **HF-HD** | Dry weight (Kg) | 61.15 | 60.10 | -1.05 | 0.84 |
| **HF-HD** | Phosphates (mg/dL) | 4.80 | 5.10 | 0.30 | 0.27 |
| **HF-HD** | Protein (gr/dl) | 6.30 | 6.60 | 0.30 | 0.23 |
| **HF-HD** | QB (ml/min) | 300.00 | 280.00 | -20.00 | 0.31 |
| **HF-HD** | Transferrin (mg/dL) | 205.00 | 194.00 | -11.00 | 0.94 |
| **HF-HD** | Treatment time (min) | 240.00 | 240.00 | 0.00 | 0.32 |
| **HF-HD** | TSAT (%) | 23.00 | 18.70 | -4.30 | 0.41 |
| **HF-HD** | Urea (mg/dL) | 158.00 | 163.00 | 5.00 | 0.59 |

Hdx, expanded hemodialysis, HFR, hemodiafiltration with reinfusion of the endogenous ultrafiltrate, OL-HDF, online hemodiafiltration, HF-HD, high-flux hemodialysis. FLC, free light chains. Hb, hemoglobin. TSAT, transferrin saturation. ERI; Erythropoietin resistance index. CRP, C reactive protein. QB, blood flow. Qconv, convective volume.
