# Supplementary material for: Uremic toxins removal and iron status: a medium-term comparison between 4 dialysis techniques (EMPIRE study)
Source: Ren Fail. 2025 May 5;47(1):2497491. doi: 10.1080/0886022X.2025.2497491 (PMC12054563; doi:10.1080/0886022X.2025.2497491)
Supplement: Table 3 Supplementary Material .docx [file IRNF_A_2497491_SM8554.docx]

Table 3 Supplementary Material: laboratory and clinical parameters for those patients who underwent HDx treatment for 48 weeks. The data are reported as median and interquartile range.

|  | **T0** | **T12** | **T24** | **T48** | **p** |
| --- | --- | --- | --- | --- | --- |
| Urea (mg/dL) | 145(134.5-186) | 135(109.5-157.5) | 138 (118.5-170.5) | 122 (105.5-139) | **0.016** |
| Creatinine (mg/dL) | 9.4 (8.2-11.5) | 9.7 (8-11.6) | 10 (8.2-11.5) | 9.8 (7.9-10.6) | 0.52 |
| Phosphates (mg/dL) | 6 (4.6-6.8) | 5.8 (4.3-7.2) | 5.4 (4.4-6.5) | 4.9 (4.5-6.5) | 0.79 |
| β2-microglobulin (mg/L) | 30.8 (27.4-39.7) | 29.6 (28.2-33.5) | 31.5 (30.1-41) | 31.2 (27.8-36.7) | 0.29 |
| *κ-FLC* (mg/L) | 176.3 (135.3-225) | 179.7 (124.7-210.1) | 190.6 (141.7-230.4) | 181.2 (140.5-213.8) | 0.28 |
| *λ -FLC (mg/L)* | 110 (72.7-142.2) | 115.7 (103.9-140) | 116 (99.7-170.7) | 107.5 (83.7-148.2) | 0.90 |
| Albumin (gr/dL) | 3.8 (3.6-3.9) | 3.6 (3.5-3.8) | 3.6 (3.5-3.8) | 3.5 (3.4-3.9) | 0.29 |
| Hb (gr/dl) | 10.5 (9.7-11.8) | 10.3 (9.9-11.1) | 11.1 (10.1-12.2) | 11.6 (10.7-11.6) | 0.45 |
| Ferritin (ng/dL) | 128 (44.5-229) | 122 (58-231.5) | 188 (87.5-326.5) | 257 (101.5-392.5) | 0.46 |
| Transferrin (mg/dL) | 175 (172-207) | 176 (147-202) | 178 (157-187) | 174 (140-190) | 0.06 |
| TSAT (%) | 16.5 (13-25) | 17.4 (15.2-23.9) | 25.2 (18.2-28.8) | 25.5 (23-30.2) | **0.001** |
| Iron dose (mg/week) | 62.5 (31.3-62.5) | 62.5 (62.5-125) | 100 (81.3-193.8) | 100 (31.3-100) | 0.10 |
| ERI | 25.2 (14.9-32.7) | 18.4 (13.6-40.6) | 25.2 (6.9-38.9) | 6.5 (0.6-37.7) | 0.56 |
| CRP (mg/dL) | 0.3 (0.1-1.3) | 0.2 (0.1-1.3) | 0.3 (0.1-2.1) | 0.3 (0.1-1.3) | 0.15 |
| KT/V | 1.4 (1.19-1.5) | 1.3 (1.0-1.6) | 1.3 (1.0-1.6) | 1.5 (1.2-1.6) | 0.83 |
| QB (ml/min) | 300 (290-300) | 300 (300-300) | 300 (290-300) | 300 (300-300) | 0.27 |
| Dialysis length (min) | 240 (240-240) | 240(240-240) | 240 (240-240) | 240 (240-240) | 0.39 |
| Ultrafiltration (L/session) | 2.5 (2.3-3) | 3 (2.1-3.6) | 2.8 (1.9-3.5) | 3(2.6-3.6) | 0.69 |
| Dry weight (Kg) | 74.8 (70.5-77.2) | 75 (70-76.9) | 74.8 (70.6-75.8) | 74.4 (69.6-75.7) | 0.43 |

HDx, expanded hemodialysis. FLC, free light chains. Hb, hemoglobin. TSAT, transferrin saturation. ERI; Erythropoietin resistance index. CRP, C reactive protein. QB, blood flow.
