# Supplementary material for: Uremic toxins removal and iron status: a medium-term comparison between 4 dialysis techniques (EMPIRE study)
Source: Ren Fail. 2025 May 5;47(1):2497491. doi: 10.1080/0886022X.2025.2497491 (PMC12054563; doi:10.1080/0886022X.2025.2497491)
Supplement: Table_4_Supplementary_Material.docx [file IRNF_A_2497491_SM8553.docx]

Table 4 Supplementary Material: laboratory and clinical parameters for those patients who underwent OL-HDF treatment for 48 weeks. The data are reported as median and interquartile range.

|  | **T0** | **T12** | **T24** | **T48** | **p** |  |
| --- | --- | --- | --- | --- | --- | --- |
| Urea (mg/dL) | | 144 (103.5-159) | 121 (90.5-150) | 138 (108.5-160) | 146 (91.5-168) | 0.74 |
| Creatinine (mg/dL) | | 8.1 (5.1-10.6) | 8.8 (4.5-10.2) | 9.3 (7.4-11.2) | 9.2 (5.8-11.4) | 0.35 |
| Phosphates (mg/dL) | | 4.6 (3.7-6) | 4.7 (4-5.6) | 3.6 (3-6.3) | 5.4 (3.9-7) | 0.55 |
| β2-microglobulin (mg/L) | | 31 (26.1-34.9) | 35 (25.4-36.6) | 33.4 (30.6-36.7) | 38.5 (33.4-43.6) | **0.002** |
| *κ−FLC* (mg/L) | | 118.4 (97.8-257.3) | 149.5 (99.2-230) | 200.6 (118.3-261.2) | 189.3 (140.1-280.1) | 0.25 |
| *λ -FLC (mg/L)* | | 121.9 (91.3-127.6) | 112.3 (89.2-128.9) | 117.3 (84.4-160.9) | 145.7 (107.1-201.8) | 0.18 |
| Albumin (gr/dL) | | 3.5 (3.1-3.7) | 3.6 (3.2-3.9) | 3.5 (3.2-3.8) | 3.2 (2.9-3.7) | 0.53 |
| Hb (gr/dl) | | 10.4 (9.4-11.4) | 10.9 (10.5-12.6) | 10.6 (10-11.7) | 10.3 (9.7-12.1) | 0.49 |
| Ferritin (ng/dL) | | 228 (85.5-417) | 200 (51-419.5) | 96 (42-285) | 114 (45-344.5) | 0.21 |
| Transferrin (mg/dL) | | 183 (147.5-211) | 183 (156-221) | 165 (157.5-228.5) | 182 (124-247) | 0.76 |
| TSAT (%) | | 20 (7.6-26.5) | 20.6 (12.8-27.6) | 15.4 (12.8-29.1) | 11.4 (9.2-22.7) | 0.29 |
| Iron dose (mg/week) | | 0 (-112.5) | 62.5 (0-156.3) | 0 (0-156.3) | 62.5 (0-112.5) | 0.82 |
| ERI | | 21.5 (13.5-46.2) | 8.4 (0-47.2) | 21.8 (12.5-43.7) | 31.6 (9.1-46.1) | 0.34 |
| CRP (mg/dL) | | 0.56 (0.2-7.9) | 0.5 (0.3-0.8) | 0.42 (0.2-0.9) | 0.7 (0.4-4.7) | 0.66 |
| KT/V | | 1.4 (1.2-1.6) | 1.5 (1.3-1.6) | 1.5 (1.2-1.8) | 1.4 (1.4-1.7) | 0.96 |
| QB (ml/min) | | 300 (270-300) | 300 (280-300) | 300 (275-300) | 300 (265-315) | 0.82 |
| Dialysis length (min) | | 240 (210-240) | 240 (210-240) | 240 (210-240) | 240 (240-240) | 0.10 |
| Qconv (L/ session) | | 20.5 (19.5-21.5) | 20.3 (19.7-21.5) | 20.5 (19.7-21.9) | 21.1 (18.4-23.2) | 0.17 |
| Ultrafiltration (L/session) | | 2.5 (2-3) | 2.5 (2.3-3) | 2.5 (1.9-3) | 2.5 (2.3-2.9) | 0.61 |
| Dry weight (Kg) | | 67 (56.8-82.8) | 67 (57.7-80.2) | 69.1 (56.8-81.5) | 72.3 (56.9-85.2) | 0.74 |

OL-HDF, online hemodiafiltration. FLC, free light chains. Hb, hemoglobin. TSAT, transferrin saturation. ERI; Erythropoietin resistance index. CRP, C reactive protein. QB, blood flow. Qconv, convective volume.
