# Supplementary material for: Uremic toxins removal and iron status: a medium-term comparison between 4 dialysis techniques (EMPIRE study)
Source: Ren Fail. 2025 May 5;47(1):2497491. doi: 10.1080/0886022X.2025.2497491 (PMC12054563; doi:10.1080/0886022X.2025.2497491)
Supplement: Table 2 Supplementary Material.docx [file IRNF_A_2497491_SM8552.docx]

Table 2 Supplementary Material: laboratory and clinical parameters for those patients who underwent HFR-Aeq treatment for 48 weeks. The data are reported as median and interquartile range.

|  | **T0** | **T12** | **T24** | **T48** | **p** |  |
| --- | --- | --- | --- | --- | --- | --- |
| Urea (mg/dL) | | 129 (98-149.5) | 115 (98-134) | 121 (101.5-145.5) | 116 (86.5-142) | 0.52 |
| Creatinine (mg/dL) | | 8.7 (7.1-10.2) | 8.4 (7.3-11.9) | 8.9 (8.1-10.5) | 9.1 (8.3-9.8) | 0.17 |
| Phosphates (mg/dL) | | 4.8 (4.5-5.6) | 5.2 83.8-6.5) | 5.2(4.4-5.9) | 5.7 (3.8-6.6) | 0.62 |
| β2-microglobulin (mg/L) | | 32.6 (30-36.9) | 36.7 (32.5-40) | 38.7 (30.1-44.2) | 34.8 (30.2-36.8) | 0.27 |
| κ-FLC (mg/L) | | 168.8 (140.2-218.8) | 151.8 (128.5-166.4) | 152.9(126.9-196.1) | 146.1 (114.9-189.6) | 0.30 |
| λ -FLC (mg/L) | | 98.7 (79.4-173.6) | 96.8 (80.8-162.8) | 100.4 (78.9-169.7) | 100.6 (72.6-158.3) | 0.83 |
| Albumin (gr/dL) | | 3.4 (3.1-3.5) | 3.5 (3.2-3.7) | 3.6 (3.3-3.8) | 3.4 (3.3-3.5) | **0.03** |
| Hb (gr/dl) | | 10.1 (9.7-11.6) | 10.5(9.9-12.1) | 11.1 (10.2-12.3) | 10.5 (9.2-11.9) | 0.55 |
| Ferritin (ng/dL) | | 123 (74.5-231.5) | 158 (86-246.5) | 133 (64-538.5) | 131 (43.5-487.5) | 0.76 |
| Transferrin (mg/dL) | | 209 (145.5-244.5) | 169 (160-223) | 184 (161.5-238.5) | 186 (157.5-219.5) | 0.27 |
| TSAT (%) | | 16.5 (13.3-29.9) | 21.3 (16.6-29.5)0 | 19.1 (16.3-31.7) | 19.7 (14.1-26.6) | 0.81 |
| Iron dose (mg/week) | | 100 (31.2-200) | 200 (50-300) | 100 (0-300) | 187.5 (50-300) | 0.28 |
| ERI | | 17.9 (10-32.9) | 20.1 (11.6-27.5) | 8.8 (3.7-28.6) | 18.9 (19.4-35.9) | 0.15 |
| CRP (mg/dL) | | 0.8 (0.3-1.2) | 0.5 (0.2-1.1) | 0.3 (0.2-1.2) | 0.5 (0.2-1.8) | 0.38 |
| KT/V | | 1.53 (0.9-1.7) | 1.3 (1.1-1.6) | 1.5 (1.0-1.5) | 1.3 (1.0-1.7) | 0.90 |
| QB (ml/min) | | 300 (280-350) | 300 (300-350) | 300 (300-325) | 300 (300-300) | 0.93 |
| Dialysis length (min) | | 240 (225-240) | 240 (240-240) | 240 (225-240) | 240 (225-240) | 0.60 |
| Ultrafiltration (L/session) | | 2.9 (2-3.2) | 2.5 (1.8-2.9) | 2.4 (2.1-2.9) | 2.7 (2.2-3.2) | 0.35 |
| Dry weight (Kg) | | 71.7 (52.1-89.8) | 70.7 (52.1-91) | 69.9 (51.6-91.9) | 66.9 (53.3-89.5) | 0.11 |

HFR, hemodiafiltration with reinfusion of the endogenous ultrafiltrate. FLC, free light chains. Hb, hemoglobin. TSAT, transferrin saturation. ERI; Erythropoietin resistance index. CRP, C reactive protein. QB, blood flow.
