# Supplementary material for: Uremic toxins removal and iron status: a medium-term comparison between 4 dialysis techniques (EMPIRE study)
Source: Ren Fail. 2025 May 5;47(1):2497491. doi: 10.1080/0886022X.2025.2497491 (PMC12054563; doi:10.1080/0886022X.2025.2497491)
Supplement: Table 5 Supplementary Material.docx [file IRNF_A_2497491_SM8551.docx]

Table 5 Supplementary Material: laboratory and clinical parameters for those patients who underwent HF-HD treatment for 48 weeks. The data are reported as median and interquartile range.

|  | **T0** | **T12** | **T24** | **T48** | **p** |  |
| --- | --- | --- | --- | --- | --- | --- |
| Urea (mg/dL) | | 158 (129.5-177.5) | 163 (124-172) | 153 (126-181) | 151 (123.5-190.5) | 0.95 |
| Creatinine (mg/dL) | | 7.8 (6.3-9.2) | 8.9 (7.3-9.7) | 9 (6.9-9.3) | 8.4 (7.6-10.6) | 0.44 |
| Phosphates (mg/dL) | | 4.8 (3.8-5.9) | 5.1 (4.6-6.2) | 5.5 (4.6-6.8) | 5 (4.4-7.1) | 0.29 |
| β2-microglobulin (mg/L) | | 30.4 (18.8-35.3) | 33.1 (23.9-35.9) | 33.4 (20.7-42.1) | 35.6 (21.9-37.6) | 0.30 |
| *κ-FLC* (mg/L) | | 112.6 (80.1-169.3) | 116.6 (94.1-215.6) | 119.5 (107.9-122.3) | 122.9 (74.3-193) | 0.61 |
| *λ -FLC (mg/L)* | | 91 (61.3-134.6) | 79.7 (62 - 171) | 99.2 (80.3-140.2) | 128.3 (62.6-193.1) | 0.32 |
| Albumin (gr/dL) | | 3.8 (3.6-4) | 3.8 (3.7-4.3) | 3.7 (3.5-4) | 3.7 (3.5-4) | 0.34 |
| Hb (gr/dl) | | 11.1 (10.1-11.9) | 11.5 (9.5-12.1) | 11.1 (9.9-11.7) | 11.5 (9.9-13.2) | 0.73 |
| Ferritin (ng/dL) | | 113 (43-349.5) | 216 (57.5-268.5) | 297 (62-475) | 276 (111.5-335) | 0.93 |
| Transferrin (mg/dL) | | 205 (157.5-241) | 194 (160.5-229.5) | 192 (162-223) | 192 (133-215) | 0.28 |
| TSAT (%) | | 23 (13.7-39.1) | 18.7 (14.7-27.1) | 18.4 (12.4-52.2) | 18.9 (11.8-28) | 0.37 |
| Iron dose (mg/week) | | 0 (0-112.5) | 0 (0-156.3) | 0 (0-187.3) | 0 (0-200) | 0.86 |
| ERI | | 11.3 (0-49.8) | 20.3 (8.6-32.9) | 14.8 (0-32) | 12.8 (4.9-38.9) | 0.45 |
| CRP (mg/dL) | | 0.3 (0.1-1.1) | 0.5 (0.1-1.6) | 0.5 (0.1-1.1) | 1.1 (0.2-1.6) | 0.62 |
| KT/V | | 1.3 (1.1-1.8) | 1.5 (1.3-1.7) | 1.4 (1.3-1.8) | 1.4 (1.1-1.5) | 0.21 |
| QB (ml/min) | | 300 (260-300) | 280 (250-300) | 300 (300-300) | 300 (300-3009 | 0.20 |
| Dialysis length (min) | | 240 (225-240) | 240 (240-240) | 240 (225-240) | 240 (240-240) | 0.75 |
| Ultrafiltration (L/session) | | 2 (1.4-2.5) | 2.4 (2-2.7) | 2.3 (1.8-2.6) | 2.6 (2.2-3.1) | 0.50 |
| Dry weight (Kg) | | 61.2 (53.9-70.1) | 62.2 (54.3-69.4) | 60 (54.5-68.9 | 61.6 (51.3-70.9) | 0.75 |

HF-HD, high flux hemodialysis. FLC, free light chains. Hb, hemoglobin. TSAT, transferrin saturation. ERI; Erythropoietin resistance index. CRP, C reactive protein. QB, blood flow.
